# A comparative analysis of parechovirus protein structures with other picornaviruses

**DOI:** 10.1098/rsob.210008

**Published:** 2021-07-28

**Authors:** Aušra Domanska, Sergey Guryanov, Sarah J. Butcher

**Affiliations:** Faculty of Biological and Environmental Sciences, Molecular and Integrative Bioscience Research Programme, and Helsinki Institute of Life Sciences–Institute of Biotechnology, University of Helsinki, FI-00014 Helsinki, Finland

**Keywords:** picornavirus, parechovirus, structure, RNA stem-loop, function

## Abstract

Parechoviruses belong to the genus *Parechovirus* within the family *Picornaviridae* and are non-enveloped icosahedral viruses with a single-stranded RNA genome. Parechoviruses include human and animal pathogens classified into six species. Those that infect humans belong to the *Parechovirus A* species and can cause infections ranging from mild gastrointestinal or respiratory illness to severe neonatal sepsis. There are no approved antivirals available to treat parechovirus (nor any other picornavirus) infections. In this parechovirus review, we focus on the cleaved protein products resulting from the polyprotein processing after translation comparing and contrasting their known or predicted structures and functions to those of other picornaviruses. The review also includes our original analysis from sequence and structure prediction. This review highlights significant structural differences between parechoviral and other picornaviral proteins, suggesting that parechovirus drug development should specifically be directed to parechoviral targets.

## Graphical abstract

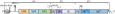

## 1. Introduction

Parechoviruses belong to a single genus within a large family, *Picornaviridae*, which comprise small, icosahedral, non-enveloped, single-stranded RNA (ssRNA) viruses approximately 30 nm in diameter. The *Parechovirus* genus is currently divided into six species *Parechovirus A*-*F* (PeV-A-F) (table 1) out of which PeV-A contains human parechoviruses. Based on the region of the genome coding for the capsid protein VP1 and VP1's antigenicity, parechoviruses from PeV-A have been divided into 19 types [7,13]. According to the national enterovirus surveillance programs implemented in a number of countries, PeV-A1 is the most prevalent human parechovirus type globally, followed by PeV-A3 and PeV-A4 [14]. Other human parechovirus types are less frequently reported, mainly in Africa and South America. Human parechoviruses cause infections ranging from asymptomatic or mild to severe illnesses, predominantly in neonates and young children [14]. The most severe symptoms are frequently associated with PeV-A3 infections in neonates, manifesting in sepsis-like disease and central nervous system infections. PeV-A3 has caused three consecutive epidemics in Australia [15]. As of yet, there are no treatments available for parechoviral infections. Parechoviruses have also been detected in other vertebrates, having been isolated from bank voles and gulls (PeV-B), rodents (PeV-C), ferrets, bats (PeV-D) and falcons (PeV-E) [2–5,16,17]. Parechovirus in geckos (PeV-F) has been identified in a large meta-transcriptomic survey [6]. There is very little information on parechoviruses that belong to PeV-B-F species.

**Table 1.** Protein sequences used for sequence analysis.

species	virus name	isolate/protein	accession number	reference
**parechoviruses**				
*Parechovirus A* (PeV A)	Human parechovirus 1 (HPeV1 or PeV-A1)	Human parechovirus 1 strain Harris	YP_009505617	[1]
*Parechovirus B* (PeV B)	Ljungan virus 1 (LV1 or PeV-B1)	Ljungan virus strain 87–012	NP_647602	[2]
*Parechovirus C* (PeV C)	Sebokele virus 1 (SEBV1)	An/B/1227/d	YP_008083730	[3]
*Parechovirus D* (PeV D)	ferret parechovirus 1 (FePeV1)	ferret/MpPeV1/NL	YP_009361997	[4]
*Parechovirus E* (PeV E)	falcon parechovirus 1 (FaPeV)	falcon/HA18_080/2014/HUN	YP_009423853	[5]
*Parechovirus F* (PeV F)	gecko parechovirus 1 (GPeV)	Yili Teratoscincus roborowskii picornavirus 2 strain LPWC210215	AVM87411	[6]
unassigned	*Rattus tanezumi* parechovirus (RtPeV)	rat/Wencheng-Rt386–3/China/2012	MF352429	unpubl., [7]
**enteroviruses**				
*Enterovirus A* (EV A)	coxsackievirus A16 (CVA16)	CA16/GD09/24	AGC82916	[8]
*Enterovirus C* (EV C)	poliovirus 1 (PV1)	Mahoney (Ohio/41)	CAA24461	[9]
**aphthoviruses**				
*Foot-and-mouth disease virus* (FMDV)	Foot-and-mouth disease virus C (FMDV C)	rp99	CAB60265	[10]
**cardioviruses**				
*Cardiovirus A*	encephalomyocarditis virus 1 (EMCV1)	Mengo Rz-pMwt	ABB97066	[11]
**human**				
*Homo sapiens*	—	PLAAT3	NP_001121675	[12]

Most of our understanding on picornavirus structure and infectious cycle comes from the remarkable efforts put into research on poliovirus and other enteroviruses [18,19]. The rest of the *Picornaviridae* family members, including parechoviruses, have received much less attention. In this review, we summarize current knowledge on the structure of the parechovirus virion as well as the structure and function of the viral proteins, highlighting similarities and differences with other picornaviruses. Cryo-EM and X-ray data on the mature human parechoviruses PeV-A1 and PeV-A3, as well as PeV-B1 enabled detailed characterization of the viral capsid; however, there are no structural data available for parechoviral non-structural proteins [14,20–23]. For better understanding of the structure and function of these proteins, we performed amino acid sequence analysis and homology modelling. First, we aligned amino acid sequences of non-structural proteins from six parechovirus isolates, each from different species, and from an unassigned *Rattus tanezumi* parechovirus (RtPV) (table 1). Then, these sequences were compared to the sequences of the homologous proteins for which molecular models are available in the Protein Data Bank (PDB). Homologous proteins, of both viral and non-viral origin, were found using the Basic Local Alignment Search Tool (BLAST) against proteins deposited in the wwPDB and further verified by multiple sequence alignments with the MUSCLE algorithm in the UGENE software suite (table 2) [21,24–28]. The sequence comparison, based on the reference sequences in table 1, was used as the basis for structuring the review, going through protein-by-protein. Parechoviruses exhibit at least six distinct features at both structural and functional level which are different from that of many other picornaviruses, which we will discuss in detail later, but summarize here. (i) VP0 is not cleaved nor myristoylated in parechoviruses [29,30]. (ii) A lipid factor, present in the hydrophobic pocket in VP1 of many enteroviruses, is absent from the parechovirus capsids [20–23]. (iii) The interactions between multiple packaging signals in genomic ssRNA and capsid proteins occurs at different sites in parechoviruses compared to enteroviruses [31,32]. (iv) The parechovirus 2A protein is homologous to eukaryotic phospholipid-metabolizing enzymes [33]. (v) Parechoviruses do not cause protein synthesis shut-off during virus replication described for enteroviruses [34]. (vi) As opposed to the guanidine hydrochloride sensitive 2C protein from enteroviruses, parechovirus infection is resistant to guanidine hydrochloride, revealing a functional difference between parechovirus and enterovirus 2C proteins [35].

**Table 2.** Identity percentage of indicated parechoviral non-structural protein sequences to reference sequences in pairwise alignment (gaps not counted).

species	2A^H-NC^		2B		2C		3A		3C^pro^		3D^pol^	
	identity to PeV A	identity to PLAAT3	identity to PeV A	identity to PV1	identity to PeV A	identity to PV1	identity to PeV A	identity to PV1	identity to PeV A	identity to PV1	identity to PeV A	identity to PV1
PeV A	100	20.7	100	12.3	100	25.2	100	11.1	100	17.0	100	24.9
PeV B	45.1	19.7	45.7	15.9	49.5	23.7	26.2	10.8	47.2	18.7	49.8	27.7
PeV C	46.1	21.3	51.1	15.3	49.8	23.4	21.0	11.3	41.0	22.7	47.9	30.2
PeV D	38.0	20.3	35.3	9.8	40.2	24.8	20.0	10.9	39.7	19.6	42.0	26.1
PeV E	45.8	21.8	45.1	14.8	51.4	24.5	25.6	11.6	51.0	20.3	46.9	25.8
PeV F	40.4	16.3	38.3	10.5	41.2	23.6	16.0	9.6	32.3	21.0	37.3	25.8
RtPV	46.8	19.9	48.9	13.9	52.1	24.7	21.5	10.7	50.5	20.3	50.0	26.6

## 2. Common features in parechoviruses and other picornaviruses

**Figure 1.**
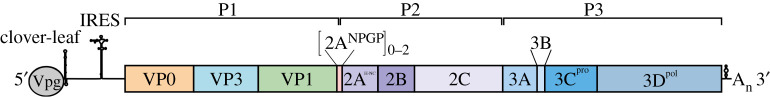
Schematic representation of parechovirus genome. Genomic ssRNA has viral protein VPg attached to its 5′ end. 5′ UTR contains clover-leaf and IRES elements necessary for RNA replication and protein synthesis initiation. The 3′ UTR contains a stem-loop structure important for virus replication as well as a polyA-tail. Virus protein-coding regions are highlighted with boxes. P1, P2 and P3 coding regions are delineated above. 2A^NPGP^ protein region is shown in brackets to indicate its presence in 0–2 non-identical copies.

**Figure 2.**
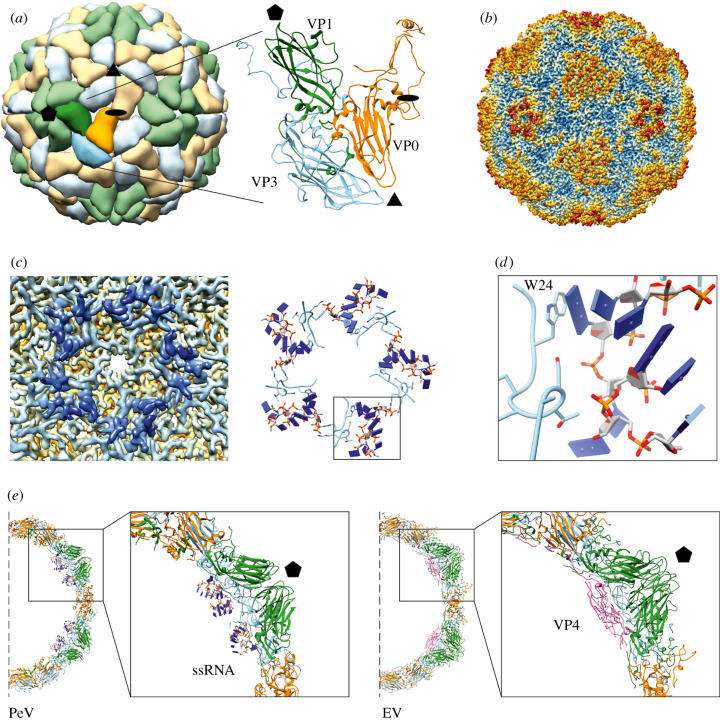
Structural details of parechovirus capsid. (*a*) Schematic view of parechovirus capsid down a twofold axis of symmetry. The icosahedral capsid consists of 60 protomers, each composed of structural proteins VP0 (orange), VP1 (green) and VP3 (light blue). The enlarged view presents modelled structural proteins for PeV-A3 (PDB ID: 6GV4). Pentagon, triangle and oval indicate fivefold, threefold and twofold symmetry axes, respectively. (*b*) Radially colour coded (steel blue, 130 Å; sky blue, 135 Å; khaki, 140 Å; orange, 145 Å; firebrick, 150 Å) surface presentation of PeV-A3 capsid resolved by cryo-EM (EMD-0069), antibody fragment density subtracted using UCSF Chimera [41]. Capsid view is the same as in panel (*a*). (*c*) Ordered ssRNA at the inner surface of the PeV-A3 capsid beneath the fivefold vertices (EMD-0069). Radially colour coded inner surface of the capsid (blue, 100 Å; light blue, 115 Å; khaki, 130 Å; orange, 140 Å; firebrick, 150 Å) and modelled ssRNA stretches (blue slabs for bases) in contact with VP3 N-terminal residues Leu16 to Arg26 (light blue) viewed down fivefold symmetry axis (PDB ID: 6GV4). Boxed segment is enlarged and slightly rotated in (*d*) to show RNA base stacking against VP3 Trp24 side chain (W24). (*e*) Comparison of atomic models of PeV-A3 (PDB ID: 6GV4) and coxsackievirus B3 (CVB3) (PDB ID: 1COV). Halves of central cross-sections of PeV-A3 and CVB3 atomic models are shown. Boxed areas show ordered ssRNA in PeV-A3 (blue slabs for RNA bases) and myristoylated VP4 in CVB3 beneath the fivefold vertices. EV, enterovirus.

Picornavirus genome organization and overall capsid structure are conserved. They have a positive-sense, ssRNA genome with a covalently linked genome-linked viral protein (VPg) at the 5′-untranslated region (UTR). The picornavirus genomic RNA consists of a single open reading frame (ORF) flanked by 5′- and 3′-UTRs. The start of the 5′-UTR is predicted to fold into a clover-leaf structure important for the replication, which is followed by an internal ribosome entry site (IRES) controlling the translation [36,37]. The 3′-UTR contains a poly-A tail resembling the messenger RNA in the host cell. The genome also possesses a cis-active RNA element (CRE), which acts as a template for VPg uridylylation, a key step in protein-primed RNA replication and transcription [38,39]. The location of CRE sites in the picornavirus genome varies, for example, poliovirus CRE is in the 2C while human parechovirus CRE is found in the VP0 coding sequence [40]. The picornavirus ORF is translated into a single polyprotein with the P1 region encoding structural proteins, followed by the P2 and P3 regions encoding non-structural proteins (figure 1). The polyprotein is cleaved by viral encoded protease(s) yielding functional proteins. The structural proteins form the protein capsid of the virion with *T* = 1, quasi *T* = 3 icosahedral symmetry. The major structural proteins of picornaviruses have a common jelly-roll fold formed by eight antiparallel β-strands arranged in two four-stranded β-sheets (figure 2*a*, inset) [42].

## 3. Parechoviral structural proteins and genomic ssRNA

### 3.1. Capsid structure

Icosahedral capsids of picornaviruses are composed of 60 protomers each made of 3 or 4 structural proteins. Five of the protomers assemble into a pentamer, 12 of which enclose genomic ssRNA to form the complete capsid (figure 2*a*). Some of the most prominent features of picornavirus capsids include star-shaped protuberances at fivefold axes that are surrounded by depressions and a propeller-like protrusions at the threefold axes (figure 2*b*) [43]. Proteins forming the protomers in parechoviruses are VP0, VP1 and VP3 (289, 231 and 253 amino acids in PeV-A1, respectively). In contrast with enteroviruses, in parechoviruses VP0 is not cleaved into VP2 and VP4 [30]. Among other picornaviruses where VP0 has been shown to remain uncleaved, only the Aichi virus (genus *Kobuvirus*) capsid structure has been published [44,45]. In enteroviruses, as the best-studied picornaviruses, VP0 cleavage into VP2 and VP4 is linked to N-terminal myristoylation of VP0, which does not occur in parechoviruses [29]. It is unclear whether VP0 from Aichi virus is myristoylated or not but it possesses a classical myristoylation motif Gxxx(S/T), where x stands for any amino acid [45]. In the assembled parechovirus virion, copies of structural protein VP1 are located around the fivefold axes, whereas VP0 and VP3 alternate around the threefold axes (figure 2*a*). The short helices from two VP0 molecules meet at each twofold axis [20–23].

As mentioned above, the capsid proteins of all picornaviruses possess eight-stranded β-barrels [42] (figure 2*a*). The structural differences among different picornavirus capsids are determined by the loops between the β-strands as well as by the capsid proteins' C- and N-termini. High-resolution structures of parechoviruses solved by X-ray crystallography and cryo-EM revealed structural differences with other picornaviruses [20–23,46]. The surface exposed loops in parechovirus capsid proteins are shorter compared to other picornaviruses leading to the formation of a shallow depression around the fivefold axis, known as a ‘canyon’ in enteroviruses [42]. Furthermore, in PeV-B1, the longer C-termini of VP1 (297 amino acids) make distinct protrusions on the surface around the fivefold axes [2,21]. What is more, most enteroviruses possess a ‘pocket’ within the VP1 β-barrel core, which is occupied by a fatty acid molecule known as a pocket factor [42]. The pocket factor can be displaced by small-molecule antivirals, such as pleconaril or WIN compounds, which leads to capsid stabilization and thereby inhibition of viral infectivity [42,47]. Parechoviruses are not able to bind the pocket factor because the corresponding space is occupied by bulky amino acid side chains [20,21,23].

### 3.2. Genomic ssRNA

Notably, in all parechovirus three-dimensional reconstructions with icosahedral averaging, extensively ordered regions of viral RNA (up to 20% of the genome) were identified in the capsid interior beneath the fivefold vertices, but not in any other picornavirus (figure 2*c*) [20–23]. The longest stretch of modelled viral RNA inside the parechovirus capsid is eight-nucleotide long, resolved in the reconstruction of PeV-A3 in complex with antibody fragments [22]. The stretch of ssRNA is anchored to the capsid via stacking interactions between a purine base and the side chain of Trp24 from VP3 (numbering for PeV-A3 A308/99) (figure 2*d*). Additional interactions of viral RNA with residues from VP1 and VP3, many of which are aromatic or positively charged, stabilize the RNA-capsid network at the inner surface of the viral capsid. Electron density attributed to viral RNA in cryo-EM structure of PeV-B1 (EMD-6394) superimpose with good agreement on the modelled RNA stretches of PeV-A3 (PDB ID: 6GV4), along with Trp15 at the N-terminus of VP3 in PeV-B structure aligning with VP3 Trp24 in PeV-A3 [21,22]. In enteroviruses, there is no conserved Trp at the position corresponding to the Trp24 in PeV-A3 VP3. Instead, structures of many enteroviruses show from one to few nucleotides (or bases only) interacting with the Trp38 side chain from the structural protein VP2 close to the twofold symmetry axes [43,48–50]. Structural comparison between parechovirus and enterovirus capsids reveals that the site below the fivefold vertices where ordered ssRNA resides in parechoviruses is largely occupied by VP4 in enteroviruses (figure 2*e*). This may also imply the differences in the process of initial interactions between the capsid protomers and the viral ssRNA as well as in subsequent steps of virus assembly. The N-termini of capsid proteins VP1, VP2 (VP0) and sometimes VP3 (for example in Aichi virus), which are located in the virion interior, are often disordered and not seen in high-resolution structures of many picornaviruses [43,44,51]. Nevertheless, the RNA affinity purification and peptide mass fingerprinting (RCAP) experiments on PeV-A1 indicate that these disordered N-termini of capsid proteins can bind viral ssRNA in the assembled virion (VP0, VP1 and VP3) and as recombinantly expressed proteins (VP0 and VP1) [52].

Consistent with the structural data discussed above, packaging signals were found throughout the parechovirus genomic ssRNA [31]. RNA-based systematic evolution of ligands by exponential enrichment (RNA SELEX) along with bioinformatics analysis revealed multiple regions, termed packaging signals, dispersed throughout the PeV-A1 RNA genome. ssRNA sequences corresponding to these packaging signals can fold into stem-loop structures, all presenting a GxU motif in their loop essential for interaction with the capsid proteins. Virus assembly mediated by multiple packaging signals implies sequence-specific binding of RNA to capsid proteins promoting protein–protein interactions needed to build the capsid [53]. Recently, similar experiments revealed the existence of packaging signals also in enteroviruses, but the capsid-ssRNA contacts occur at VP2 Trp38 rather than at VP3 Trp24 [32].

## 4. Parechoviral non-structural proteins

### 4.1. 2A proteins

**Figure 3.**
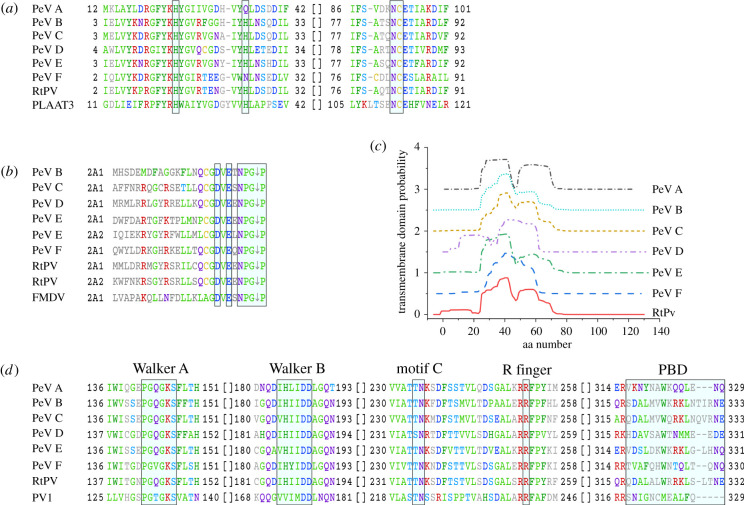
Functional motifs in 2A^H-NC^, 2A^NPGP^, 2B and 2C. (*a*) Multiple sequence alignment of regions encompassing the active site amino acid residues in phospholipase A and acyltransferase 3 (PLAAT3) with parechovirus 2A^H-NC^ proteins. Residue positions corresponding to PLAAT3 His23, His35 and NC-box are highlighted in boxes. (*b*) C-terminal sequences of 2A^NPGP^ ‘self-cleaving’ motifs from parechoviruses and foot-and-mouth disease virus aligned to the position of a break in polyprotein chain. Conserved residues of DxExNPGP motif are highlighted in boxes. (*c*) Prediction of transmembrane regions in parechovirus 2B proteins made in Phobius [62]. Plots are aligned on the *x*-axis according to protein sequence alignment. The scale of the *y*-axis is offset by 0.5. (*d*) Multiple sequence alignments of functional regions in parecho- and poliovirus 2C proteins. Conserved characteristic motifs are highlighted in boxes and labelled on top. Residues in positions with conservation over 35% are shown in colour. Sequences used in the alignment are indicated in table 1.

In terms of functions, the picornavirus 2A proteins are the most diverse proteins encoded by picornaviruses and can fall into one of at least five categories, (i) proteases, (ii) H-NC box proteins, (iii) short peptides mediating ‘self-cleavage’, (iv) unique 2A protein from hepatitis A virus with no functional motifs recognized so far and (v) unique 2A protein from cardioviruses possessing three functional motifs [54]. Many picornaviruses (e.g. enteroviruses) have 2A^pro^ protease that cleaves viral polyprotein between VP1 and 2A [55] and plays a role in shut down of host protein synthesis by cleaving several cellular proteins including eIF4G [56–58]. The 2A^H-NC^ proteins encoded by all parechoviruses and members of some other picornavirus genera including *Kobuvirus*, *Avisivirus*, *Gallivirus*, *Avihepatovirus* and *Passerivirus* have a conserved H-NC box and belong to the NlpC/P60 superfamily of proteins [54]. Proteins of NlpC/P60 superfamily are widely found across all domains of life (eukaryotes, bacteria and archaea) [59]. There are no structural data on picornaviral 2A^H-NC^ proteins, but there are structures available for other H-NC box proteins. We identified human phospholipase A and acyltransferase 3 (PLAAT3) (UniprotKB P53816), a member of NlpC/P60 superfamily, as the closest homologue to parechoviral 2A^H-NC^ in a BLAST search against wwPDB database (accessed on 17 August 2020) [28]. Then, we compared sequences of PLAAT3 and 2A^H-NC^ from isolates belonging to different parechovirus species (table 1). This analysis led us to conclude that parechovirus 2A^H-NC^ proteins are relatively conserved as non-human parechovirus 2A^H-NC^ proteins are 38–47% identical to human parechovirus 2A^H-NC^, and they all are 16–22% identical to PLAAT3 (table 2). PLAAT3 belongs to a group of phospholipid-metabolizing enzymes together with PLAAT1 (UniprotKB Q9HDD0), PLAAT2 (UniprotKB Q9NWW9) and PLAAT4 (UniprotKB Q9UL19) [60,61]. The X-ray structure of PLAAT3 presents the active site of H-NC box proteins with strictly conserved catalytic Cys113 and His23 serving as a general base (PDB ID: 4DOT) [12]. The third amino acid stabilizing the position of His23 in the catalytic reaction is His35, which is not strictly conserved and replaced by Asn in PLAAT1 [12]. Parechovirus 2A^H-NC^ proteins contain conserved Cys and His corresponding to the positions of catalytic Cys113 and His23 in PLAAT3, respectively, making the H-NC box (figure 3*a*). The parechovirus 2A^H-NC^ proteins have either His, Asn or Gln in the position corresponding to the PLAAT3 His35 (figure 3*a*). The enzymatic activity of parechovirus 2A^H-NC^, however, remains to be tested. Interestingly, during enterovirus infection, the enzymatic function of a host phospholipase PLAAT3 is implicated in viral genome release into the cytoplasm [63]. The parechovirus 2A^H-NC^ is a non-structural protein and thus its involvement in the viral RNA exit into the cytoplasm is questionable, unless a few copies of this protein are present in the virion. The parechovirus 2A^H-NC^ could possibly work at later steps in the viral infectious cycle, in line with data showing that human parechovirus 2A^H-NC^ protein binds to RNA with preferred specificity to human parechovirus 3′-UTR [64].

In the non-human parechoviruses, the 2A^H-NC^ protein is preceded in polyprotein sequence by one or more unrelated 2A^NPGP^ proteins, and in these cases, 2A proteins are designated as 2A1, 2A2 and so forth (figure 1). The C-termini of parechovirus 2A^NPGP^ proteins share the DxExNPGP ‘self-cleavage’ motif similar to cardio-, erbo-, tescho- and aphthovirus 2A^NPGP^ peptides [2] (figure 3*b*). The DxExNPGP motif mediates so-called ‘ribosome skipping’, when the peptide bond between the DxExNPG sequence and the first Pro of the following protein is not formed during polyprotein translation [65]. Although the ‘ribosome skipping’ efficiencies of 2A^NPGP^ sequences from different viruses vary, this allows rapid release of P1–2A part from the rest of the polyprotein (P2 and P3) [66]. Such self-cleavage motifs, first identified in picornaviruses, are also found in other RNA viruses [67,68].

### 4.2. 2B proteins

The RNA replication of positive-sense ssRNA viruses occurs in viral-induced compartmentalized membranes, called replication organelles, where viral replication complexes assemble [69]. It has been shown in a well-established enterovirus system that, upon infection, intracellular membrane rearrangements are triggered by non-structural viral proteins 2B, 2C, their precursor 2BC and 3A which were shown to localize in intracellular membranes [70–73]. Similarly, 2B, 2C and 3A from human parechoviruses were found in cellular membranes [74]. Enterovirus infection leads to disassembly of the Golgi complex concurrently with the assembly of replication organelles [75,76]. During enterovirus infection massive rearrangements of intracellular membranes occur [75,77,78]. By contrast, parechovirus infection leads to relatively minor changes in the intracellular membrane architecture [79]. This mild rearrangement of intracellular membranes is possibly reflected in the parechovirus insensitivity to itraconazole and naturally occurring compound OSW-1, both blocking enterovirus infection via oxysterol-binding protein 1 (UniprotKB P22059) [80,81]. Oxysterol-binding protein 1 is a lipid transporter which shuttles sterols from the endoplasmic reticulum (ER) to the Golgi complex in exchange to phosphatidylinositol 4-phosphate (PI4P) [82]. The lipid composition of virus induced organelles differs from the intracellular membranes as replication organelles are enriched in PI4P [76,83]. PI4P, a ubiquitous small lipid with regulatory functions, produced by cellular phosphatidylinositol 4-kinase beta (PI4Kbeta, UniprotKB Q9UBF8) is found in membrane compartments of all eukaryotic cells [84]. In uninfected cells, the PI4Kbeta is associated with the Golgi complex [85].

To better understand parechoviral 2B protein functions, we analysed 2B amino acid sequence conservation among selected parechovirus and poliovirus isolates (table 1). The parechovirus 2B proteins comprising 122–142 amino acids are longer than their counterparts in enteroviruses which are only 95–99 amino acids long. In pairwise amino acid sequence alignment, the non-human parechovirus 2B proteins show 35–51% sequence identity with PeV-A1 2B. When all parechovirus species are similarly compared to prototype enterovirus poliovirus 2B amino acid sequence, the parechovirus 2B proteins show low sequence identity (10–16%) (table 2). It has been shown that enterovirus 2B proteins have two transmembrane helices which insert into the membranes and increase their permeability, a feature intrinsic to viroporins [86–88]. Using an algorithm for transmembrane region predictions in parechoviral 2B proteins, we identified an extended transmembrane region sufficient to form two transmembrane helices (figure 3*c*). The predicted transmembrane helices in the case of PeV-A1 2B protein (figure 3*c*) likely correspond to the hydrophobic regions HR1 and HR2 in 2B from enteroviruses (poliovirus, CVB3 and EV-A71) indicating that parechovirus 2B proteins may act as viroporins, too [89]. In line with this, the individually expressed PeV-A1 2B protein was found to localize to the ER [74].

### 4.3. 2C proteins

The non-structural protein 2C is one of the most conserved proteins within the *Picornaviridae* family [90]. These proteins bear an ATPase domain exhibiting features similar to helicase superfamily 3 proteins and are involved in many vital processes during the viral life cycle [91,92]. Our analysis shows that 2C proteins in non-human parechoviruses are 41–51% identical to PeV-A1 2C, and parechovirus 2C proteins (329–330 amino acids long) are 23–25% identical to poliovirus 2C (table 2). In the case of cells infected with PeV-A1, the 2C protein is found in the trans-Golgi and altered ER membranes [79]. Similarly to enterovirus 2C proteins, which comprise 322–330 amino acids and localize in Golgi-derived membranes, PeV-A1 2C possesses enzymatic ATPase activity and additionally AMP kinase activity [77,93–95]. Unlike enterovirus 2C, the parechovirus 2C protein is resistant to guanidine hydrochloride, as parechovirus infection is not affected by this compound [35]. X-ray data are available for a soluble domain of 2C from poliovirus and EV-A71 [96,97]. Amino acid sequence analysis of parechovirus 2C shows a similar domain organization to that of 2C proteins in enteroviruses (figure 3*d*). The exception is a missing zinc finger domain in parechovirus 2C. The ATPase domain from both poliovirus and EV-A71 2C proteins includes canonical Walker motifs A and B as well as helicase superfamily 3-specific motif C. Motif C is followed by an arginine finger (R-finger) [96,97]. Walker motifs A, B and motif C are responsible for nucleotide binding and the R-finger is involved in modulating NTP binding or hydrolysis [91]. The zinc finger composed of 3 or 4 Cys residues coordinating a zinc atom is essential for correct overall folding of the enteroviral 2C protein [97]. The pocket-binding domain located at the very end of the 2C is thought to be important for protein oligomerization [97]. Superfamily 3 helicases function as oligomers, for example as hexamers or double hexamers [91,98]. Disrupting self-oligomerization of 2C abolishes ATPase activity [97]. Modelling has shown that in poliovirus 2C, as in numerous other AAA+ ATPases, the neighbouring protomer contributes the R-finger to the active site, explaining why 2C oligomerization is key for ATPase activity [96,98].

Notably, the parechoviral 2C proteins contribute to the severity of the symptoms of the viral infection, as shown for PeV-A3. PeV-A3 variants carrying changes from polar to basic amino acids at positions 317 and 324 in the C-terminus of 2C are more frequently associated with severe symptoms [15]. The mechanism of action related to this phenomenon is not clear yet, though these changes are located in the pocket-binding domain of 2C and thus might contribute to protein oligomerization and in turn to its activity (figure 3*d*).

### 4.4. 3A proteins

Our sequence analysis shows that parechovirus 3A proteins are significantly longer (117–130 amino acids) than their enterovirus counterparts (77–89 amino acids). In addition, the 3A proteins from non-human parechoviruses have relatively low (16–26%) sequence identity to 3A from PeV-A1 in pairwise amino acid sequence comparison. Even lower (10–12%) identity is detected between amino acid sequences of 3A proteins if all parechoviruses are compared to poliovirus (table 2). It has been shown that individually expressed 3A proteins from PeV-A1 co-localize with Golgi markers without causing large membrane alterations [74]. In addition, mammalian two-hybrid assay showed that 3A from PeV-A1 binds to a Golgi resident protein GCP60 (UniprotKB Q9H3P7) [99]. This is similar to enterovirus 3A proteins which were shown to localize in intracellular membranes and to interact with various cellular factors, including GCP60, leading to inhibition of the ER-to-Golgi vesicle transport and disassembly of the Golgi complex [99,100]. In uninfected cells GCP60, a non-integral membrane protein, resides in the Golgi apparatus and its association with the membranes is tightly controlled by the Golgi transmembrane protein Golgin subfamily B member 1 (UniprotKB Q14789) [101]. The important function of Golgi resident protein GCP60 in the cells is the recruitment of PI4Kbeta to the Golgi membranes, and stimulation of PI4P production [102]. As we discussed in the 2B protein section above, the picornaviral replication sites on the membranes are enriched in PI4P.

There are no structural data on parechovirus 3A protein, but structural studies done with other picornaviral 3A shed light on how PI4Kbeta is recruited to the viral replication organelles [103,104]. Atomic structures and computational simulations indicate that enteroviral 3A is anchored to the membrane via a C-terminal alpha-helix whereas the cytoplasmic domain wraps around the GCP60 GOLD domain [104]. This way the 3A protein recruits Golgi resident protein GCP60 to the site of virus replication, which helps then to recruit other cellular factors required for viral replication machinery. Although showing limited identity to 3A sequence from enteroviruses, parechoviral 3A protein may recruit GCP60 via a similar mechanism through interaction with the GOLD domain.

### 4.5. 3AB and 3B proteins

Similarly to 3A alone, individually expressed human parechovirus 3AB localizes in the Golgi membranes [74]. As shown for enteroviruses, in 3AB, the 3B (also known as VPg) is responsible for interaction with the 3D^pol^ polymerase [105]. To serve as a primer for 3D^pol^, however, VPg has to be released from 3AB, which is done by 3CD^pro^, precursor for both viral protease 3C^pro^ and polymerase 3D^pol^ [106,107]. VPg is a peptide of 26–33 amino acids in parechoviruses (for comparison, in poliovirus VPg is 22 amino acids long). The N-terminal part, including Tyr3, is strictly conserved in all parechovirus as well as other picornavirus VPg peptides. As shown for enteroviruses, the side chain of Tyr3 in VPg is di-uridylylated by the viral polymerase 3D^pol^ and VPg remains linked to the 5′-end of the ssRNA genome in the virion [38,108,109]. A two-molecule model for 3D^pol^ during picornavirus VPg uridylylation was suggested, based on structural as well as biochemical studies of enteroviruses and foot-and-mouth disease virus (FMDV) [108,110–112]. According to this mechanism, one molecule of 3D^pol^ binds VPg via a noncatalytic site and presents Tyr at position 3 for the uridylylation by another 3D^pol^ molecule [111,112]. There are no similar studies reported for the parechovirus system so far.

### 4.6. 3C^pro^ and 3CD^pro^ proteins

**Figure 4.**
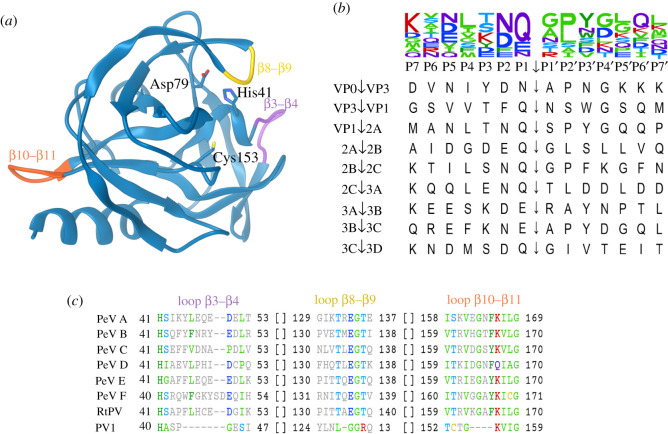
Parechovirus 3C^pro^ homology model and specificity. (*a*) Structure model of PeV-A1 Harris 3D^pro^ based on I-TASSER structure prediction [114]. Active site amino acid residues are labelled, and loops β3–β4, β8–β9 and β10–β11 are highlighted in purple, yellow and orange, respectively. (*b*) Cleavage site sequences in PeV-A1 Harris polyprotein. VP0↓VP3 and VP3↓VP1 sites have been confirmed experimentally [1]; other sites are predicted by homology. The sequence logo on top depicts amino acid residue frequency in positions around the cleavage site [115]. (*c*) Multiple sequence alignments of loop regions β3-β4, β8-β9, β10-β11 of parechovirus and poliovirus (PV1) 3C^pro^. Residues in positions with conservation over 35% are shown in colour. Sequences used in the alignment are indicated in table 1.

Parechoviruses possess only one protease 3C^pro^. Parechovirus 3C^pro^ is a chymotrypsin-like cysteine protease, 194–198 amino acids in length, classified into the PA(C) clan of peptidases together with proteases from other RNA viruses including 2A^pro^ and 3C^pro^ from picornaviruses [113]. The parechovirus 3C^pro^ presumably processes all the junctions between intermediate and mature peptides in the virus polyprotein apart from ‘self-cleaved’ 2A^NPGP^ sites. Our analysis shows that 3C^pro^ proteins from non-human parechovirus species have 32–51% sequence identity to PeV-A1 3C^pro^, while 3C^pro^ from different parechovirus species has just 17–23% sequence identity to poliovirus 3C^pro^ (table 2). To date, there is no experimentally solved three-dimensional structure of parechovirus 3C^pro^ although there are structures from a number of enterovirus 3C^pro^ proteins. We used homology-based modelling to predict three-dimensional structure of PeV-A1 3C^pro^ (figure 4*a*). As mentioned above, the 3C^pro^ proteins from picornaviruses share a two β-barrel fold characteristic for chymotrypsin (a serine protease secreted by a pancreas as proenzyme) [116]. The active site of such proteases consists of His, Asp (in parechoviruses) or Glu (in enteroviruses), and Cys catalytic triad (figure 4*a*). The 3C^pro^ uses the active site Cys as a nucleophile to break the peptide bond in the polyprotein. The imidazole ring of the His works as a general base, and Asp or Glu is important to keep the imidazole ring in the proper position for interaction with the catalytic Cys [116]. Comparison of cleavage site sequences in PeV-A1 polyprotein reveals 3C^pro^ preference for Gln, Glu or Asn in P1 position, and Gly or Ala in P1′ position (figure 4*b*). This implies relatively broad and diverse specificity of parechovirus 3C^pro^ in contrast with poliovirus 3C^pro^ which cleaves preferentially Gln–Gly bonds [117]. In addition to the active site, picornavirus 3C^pro^ has an RNA-binding site located on the opposite side of the protein molecule [118]. Importantly, picornavirus 3CD^pro^ exhibits protease activity and is known to specifically bind viral RNA [119]. The 3D^pol^ domain within the 3CD precursor modulates the protease specificity and binding to RNA sequences [120,121].

Structure prediction algorithms suggest that the PeV-A1 3C^pro^ polypeptide adopts the enterovirus 3C^pro^ structure fold (figure 4*a*). Interestingly, the predicted model features three extended loops compared to poliovirus 3C^pro^. Multiple sequence alignment of prototypic parechovirus 3C^pro^ proteins suggests that these loops are characteristic for all parechovirus species (figure 4*c*). The loop between β3 and β4 strands is adjacent to the His41 in the active site, the loop between β8 and β9 strands is spatially close to the active site, and the loop between β10 and β11 strands is part of a putative RNA-binding domain [118]. Possibly, these structural differences between enterovirus and parechovirus 3C^pro^ proteins contribute to peptide substrate and RNA-binding specificity.

### 4.7. 3D^pol^ proteins

**Figure 5.**
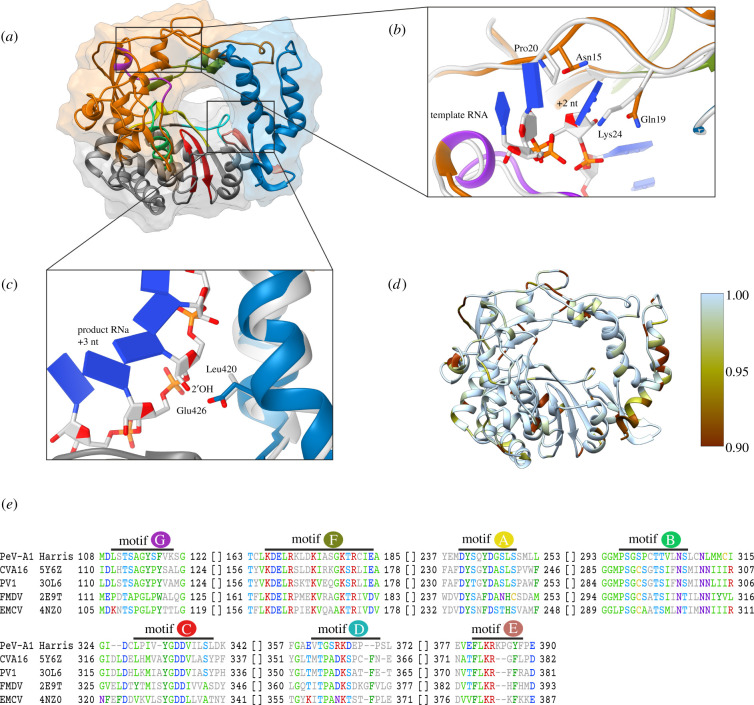
PeV-A1 3D^pol^ homology model-based structure and conservation. (*a*) Homology model and surface representation of PeV-A1 Harris 3D^pol^ based on I-TASSER structure prediction [114]. Fingers, palm and thumb domains [127] are coloured in orange, grey and blue, respectively. Conserved motifs A, B, C, D, F and G [128] are highlighted in yellow, green, red, cyan, salmon, olive green and purple, respectively. (*b*) Binding pocket for template RNA +2 nucleotide. Poliovirus (PV1) 3D^pol^ with template RNA (PDB ID: 3OL6) is shown in light grey. (*c*) Interaction with product RNA +3 nucleotide 2′OH. PV1 3D^pol^ is depicted as in b. (*d*) PeV-A1 3D^pol^ homology model coloured by amino acid conservation. (*e*) Multiple sequence alignment of conserved sequence motifs in PeV-A1 Harris and coxsackievirus A16 (CVA16), PV1, foot-and-mouth disease virus (FMDV) and encephalomyocarditis virus (EMCV) 3D^pol^ proteins. PDB IDs are given for the proteins with experimentally solved structure. Residues in positions with conservation over 35% are shown in colour.

3D^pol^ protein is an RNA-dependent RNA polymerase (RdRP) that replicates genomic viral RNA without a DNA intermediate. Parechovirus 3D^pol^ polymerase is 467–472 amino acids long and is located at the very end of the full-length viral polyprotein (figure 1). Our amino acid sequence comparisons between 3D^pol^ proteins from non-human parechoviruses show 37–50% identity to that of PeV-A1. In comparison, amino acid sequences from all parechovirus 3D^pol^ proteins have 24–30% identity to poliovirus 3D^pol^ (table 2). To date, there are X-ray or cryo-EM three-dimensional structures of RdRPs available for a plethora of positive-strand RNA viruses, but not parechoviruses [122–124]. The structural data available for positive-strand RNA virus RdRPs show a high level of conservation. All DNA and RNA polymerases, including viral RdRPs, possess canonical human right-hand architecture with palm, finger and thumb domains originally described in the structure of the DNA polymerase I Klenow fragment [125,126] (figure 5*a*). A unique feature of the RdRPs is the finger domain loops, named fingertips, which interconnect finger and thumb domains, thereby creating a ‘closed-hand’ architecture [123,124,129]. The ‘closed-hand’ architecture is not seen in other than RdRP type of polymerases [123,124,129]. The most conserved palm domain accommodates the active site of the polymerase. The finger and thumb domains interact with the template RNA [122,124,130]. The RdRP is a dynamic structure, the palm domain undergoes conformational changes upon NTP binding and the thumb domain accommodates movements allowing translocation of the template RNA [131]. Polymerases use a two-metal catalytic mechanism, in which two magnesium ions are coordinated by two aspartic acid residues located in the palm domain, the priming nucleotide 3′ hydroxyl group and the NTP triphosphate [132]. The active site closure mechanism used by the RdRP from (+)ssRNA viruses differs from (−)ssRNA viruses and is related to the high RNA replication rate and low fidelity, meaning that picornavirus polymerases introduce a high number of errors during replication (in the range of 10^−4^ per nucleotide copied) [133,134]. Low replicative fidelity leads to a population of virus variants known as quasispecies, which gives the flexibility to adapt to the changing environment [135].

We performed three-dimensional structure prediction, showing that the amino acid sequence of PeV-A1 3D^pol^ likely folds into the characteristic ‘closed-hand’ architecture found in all RdRPs (figure 5*a*). In comparison with the well-characterized poliovirus RdRP, human parechovirus 3D^pol^ potentially interacts with template and product RNAs through different mechanisms. For example in poliovirus, the binding pocket for +2 nucleotide base of template RNA is formed by Pro20 and Lys24 which correspond to Asn15 and Gln19 in human parechoviruses, respectively (figure 5*b*). Moreover, we observed significant variation in these residues when we compared 3D^pol^ sequences from different parechovirus species. In non-human parechovirus 3D^pol^ sequences the binding residues for +2 nucleotide base of template RNA is Pro15, which aligns well with poliovirus Pro20 but differs from Asn15 in PeV-A1. In regard to the second residue in the RNA template binding pocket corresponding to poliovirus Lys24, there is much more variation in non-human parechovirus 3D^pol^ sequences (Lys19, Gln19, Ala19 or Ser19). In addition, all parechovirus RdRPs have Glu426 (PeV-A1 numbering) to interact with the +3 nucleotide 2′OH of the product RNA, while in poliovirus 3D^pol^ this position corresponds to Leu420, making a hydrophobic contact with the RNA ribose cycle (figure 5*c*) [126]. Altogether human parechovirus RdRPs tend to make more H-bonds and potentially bind RNA with higher affinity. Apart from the conserved motifs, RdRPs within the human parechovirus isolates may have considerable variations in amino acid residues in certain positions, mostly on the protein surface (figure 5*d*). Furthermore, multiple sequence alignments of sequence motifs A–G [128] in parechovirus RdRP with homologues from other picornaviruses often reveal significant sequence variations (figure 5*e*).

## 5. Perspectives

RNA viruses are present in great numbers and diversity in a wide range of hosts, including vertebrates and invertebrates. RNA viruses have a significant impact not only on human health but also on agricultural industry, and they often carry a zoonotic potential. Our knowledge on RNA viruses is still growing as seen, for example, by a large number of new genera recently defined in the *Picornaviridae*. Many picornaviruses including parechoviruses were identified in large metagenomics screens of diverse host species [6,136,137]. In this review, we combined data on parechoviruses available through scientific reports and public databases, such as GenBank and wwPDB. Detailed structural information available on parechovirus virions show prominent differences with enteroviruses, the best-studied group of picornaviruses. The data on parechoviral non-structural proteins is mostly limited to nucleotide or amino acid sequences. The exception is PeV-A1, for which non-structural protein localization and some functional studies have been performed. Here, we show that in pairwise alignments to PeV-A1 proteins parechovirus 2C is the most conserved protein within *Parechovirus* genus, followed by 3D^pol^, 2B, 3C^pro^ and 2A^H-NC^ proteins (table 2). The parechoviral 3A is least conserved among non-structural proteins (table 2). When amino acid sequences of parechoviruses were compared to the corresponding sequences of poliovirus, the highest conservation was observed with 3D^pol^, 2C and 3C^pro^ (table 2). Proteins 2B and 3A show limited conservation to corresponding poliovirus amino acid sequences (table 2). The parechovirus 2A^H-NC^ protein has homologues in members of numerous *Picornaviridae* genera but not in isolates from the *Enterovirus* genus. We identified human phospholipase PLAAT3, an H-NC protein, as the closest protein to parechoviral 2A^H-NC^ for which a three-dimensional structure has been published.

Virus proteins critical for the virus infectious cycle can be used as drug targets in the fight against the diseases caused by these viruses. Nowadays, information about the three-dimensional structure of the target proteins, in addition to the knowledge of their functions, is extensively used in the identification and optimization of candidate drug molecules. Structure-based drug design became tightly integrated into the therapeutic drug development platforms since the end of the last century when X-ray crystallography, the main structural method, has been established and led to the accumulation of structural data on various biological molecules [138]. The structure-based approach has been successfully used to develop antivirals against important pathogens such as HIV and influenza virus, driving to licensed drugs against HIV-1 protease (nelfinavir) and against influenza neuraminidase (zanamivir) [139,140]. Regarding antivirals against picornaviruses, to date there are no FDA-approved drugs to treat picornavirus infections despite tremendous efforts exerted by academia and the pharmaceutical industry. These efforts, however, helped to identify a number of molecules that block picornavirus (specifically enterovirus) replication [141]. Some of the picornavirus replication inhibitors were evaluated in clinical trials, among which also capsid binders, such as pleconaril, and inhibitors of viral non-structural proteins acting on protease (rupintrivir and AG7404), polymerase (ribavirin), 2C (fluoxetine), as well as IRES-dependent translation (amantadine) [142]. In parallel to *de novo* drug development, drug repurposing has been widely adopted for finding potential FDA-approved drugs to treat other medical conditions. For example nitazoxanide, a drug licensed as antiparasitic therapy, shows broad-spectrum antiviral activity and has been repurposed for influenza treatment [143]. Furthermore, a clinical trial to evaluate the efficacy and safety of nitazoxanide in the treatment of colds caused by the enterovirus and rhinovirus infections has been recently completed, but results have not yet been published (NCT03605862). Another example is the FDA-approved anti-fungal drug itraconazole, which was also found to inhibit enterovirus, but not PeV-A1, infection [80]. Mutations that confer resistance to itraconazole map to 3A protein involved in PI4Kbeta recruitment [144].

This review highlights that potential drug targets in parechoviruses show considerable dissimilarity with their homologues from well-studied picornavirus genera including entero-, aphtho- and cardioviruses. This divergence in virus-encoded proteins translates into parechovirus insensitivity to known inhibitors of picornavirus infectious cycle, like pleconaril, itraconazole and guanidine hydrochloride. While structural data on parechovirus capsid proteins provides remarkable insight into virus assembly and neutralization by antibodies, the non-structural proteins remain to be studied in more detail.

## Data accessibility

Accession numbers for protein sequences used for the analysis are listed in table 1. PDB IDs for structural data and UniProt accession numbers for proteins are indicated in relevant figures or throughout the text.
